# TAT‐MTS‐MCM fusion proteins reduce MMA levels and improve mitochondrial activity and liver function in MCM‐deficient cells

**DOI:** 10.1111/jcmm.13435

**Published:** 2017-12-19

**Authors:** Tal Erlich‐Hadad, Rita Hadad, Anat Feldman, Hagar Greif, Michal Lictenstein, Haya Lorberboum‐Galski

**Affiliations:** ^1^ Department of Biochemistry and Molecular Biology Institute for Medical Research Israel‐Canada (IMRIC) Faculty of Medicine Hebrew University of Jerusalem Jerusalem Israel; ^2^ BioBlast‐Pharma Ltd. Tel‐Aviv Israel

**Keywords:** TAT fusion proteins, methylmalonic aciduria, methylmalonyl‐CoA mutase, mitochondria, heterologous Mitochondrial Targeting Sequences, liver secretion

## Abstract

Methylmalonic aciduria (MMA) is a disorder of organic acid metabolism resulting from a functional defect of the mitochondrial enzyme, methylmalonyl‐CoA mutase (MCM). The main treatments for MMA patients are dietary restriction of propiogenic amino acids and carnitine supplementation. Liver or combined liver/kidney transplantation has been used to treat those with the most severe clinical manifestations. Thus, therapies are necessary to help improve quality of life and prevent liver, renal and neurological complications. Previously, we successfully used the TAT‐MTS‐Protein approach for replacing a number of mitochondrial‐mutated proteins. In this targeted system, TAT, an 11 a.a peptide, which rapidly and efficiently can cross biological membranes, is fused to a mitochondrial targeting sequence (MTS), followed by the mitochondrial mature protein which sends the protein into the mitochondria. In the mitochondria, the TAT‐MTS is cleaved off and the native protein integrates into its natural complexes and is fully functional. In this study, we used heterologous MTSs of human, nuclear‐encoded mitochondrial proteins, to target the human MCM protein into the mitochondria. All fusion proteins reached the mitochondria and successfully underwent processing. Treatment of MMA patient fibroblasts with these fusion proteins restored mitochondrial activity such as ATP production, mitochondrial membrane potential and oxygen consumption, indicating the importance of mitochondrial function in this disease. Treatment with the fusion proteins enhanced cell viability and most importantly reduced MMA levels. Treatment also enhanced albumin and urea secretion in a CRISPR/Cas9‐engineered HepG2 MUT (‐/‐) liver cell line. Therefore, we suggest using this TAT‐MTS‐Protein approach for the treatment of MMA.

## Introduction

Considering the central role of mitochondria in ATP production, it is not surprising that numerous diseases and disorders are associated with mitochondrial dysfunctions and mutations, including metabolic pathologies 1, 2, 3, neurodegenerative diseases 4, brain injuries 5, deafness 6, eyesight defects 7 and many more. These disorders can be classified into three groups: 1. diseases caused by mitochondrial DNA mutations; 2. diseases caused by mitochondrial mutations in nuclear DNA encoding mitochondrial proteins; 3. pathologies caused indirectly by mitochondria, in which mitochondrial dysfunction is correlated to the pathology or leads to its deterioration. Currently, there is no clinically approved cure for mitochondrial diseases and the treatment is mostly symptomatic 8, 9. One of the several developed approaches to treat mitochondrial diseases is enzyme replacement therapy (ERT).

ERT is a treatment where patients in whom a deficient or absent enzyme is replaced by a fully functional enzyme. ERT is currently available for lysosomal diseases such as Gaucher disease 10, Fabry disease 11, Hunter syndrome 12 and glycogen storage disease type II 13. However, the inability of the administered enzymes to penetrate the blood–brain barrier severely limits this approach from being implemented in the treatment of metabolic disorders that involve the central nervous system (CNS) 14, 15. One strategy for delivering proteins into cells is to fuse them with protein transduction domains (PTD), which have demonstrated high efficiency for facilitating the internalization of both homologous and heterogeneous proteins into cells at low micromolar concentrations *in vivo* and *in vitro* 15, 16, 17. In addition, the ability to target specific intracellular sublocalizations such as the nuclei, the mitochondria and lysosomes further expands the possibilities of this drug delivery system to the development of subcellular organelle‐targeted therapy 18, 19, 20. We were the first to successfully treat a mitochondrial disease such as LAD deficiency in a mouse model, using the PTD‐Protein approach 21, 22. In this system TAT, an 11 a.a PTD peptide 23, which rapidly and efficiently can cross biological membranes 24, is fused to a MTS, followed by the mitochondrial mature protein which targets the chosen protein to the mitochondria. In the mitochondria, the TAT‐MTS is cleaved off and the native protein is fully functional 21, 22. Therefore, we suggest using this TAT‐MTS‐MCM fusion protein for the treatment of MMA.

MMA pathology is a disorder of organic acid metabolism resulting from a functional defect of MCM encoded by the MUT gene 25 or a defect in the biosynthesis of its cofactor, adenosylcobalamin (AdoCbl, vitamin B12) 26. About 60 per cent of methylmalonic acidaemia cases are caused by over 100 identified mutations in the MUT gene 27. In addition, methylmalonic acidaemia could be caused by mutations in the MMAA, MMAB or MMADHC genes, which are essential for the proper function of MCM 28. Recently, a few patients have been described with mild MMA associated with mutations of the methylmalonyl‐CoA epimerase gene (MCEE) or with neurological symptoms due to Succinate‐CoA Ligase Alpha Subunit or Succinate‐CoA Ligase ADP‐Forming Beta Subunit mutations, which code for the succinate‐CoA ligase enzyme complex 29. This rare disorder of organic acid metabolism is inherited in an autosomal recessive manner and occurs with an incidence of approximately 1:48,000–1:100,000 live births 30. Defects of the MUT gene can be divided into two subgroups: mut(0), which represents a complete loss of activity, and mut(‐), representing a residual activity in the presence of AdoCbl 30.

The MCM enzyme mediates the catabolism of the branched‐chain amino acids isoleucine and valine as well as methionine, threonine, odd‐chain fatty acids and cholesterol for entrance into the Krebs cycle *via* propionyl‐coenzyme A (CoA), which requires the isomerization of l‐methylmalonyl‐CoA to succinyl‐CoA 31. MCM is encoded in the nucleus as a 750 amino acid precursor protein and transported then into the mitochondrial matrix, where its 32 amino acid MTS is cleaved 25. The mature enzyme, 718 amino acids in size, forms a homodimer, and each subunit binds one molecule of adenosylcobalamin 32. Deficiency of the enzyme leads to accumulation of propionyl‐CoA and methylmalonyl‐CoA, which is hydrolysed to form methylmalonic acid, and is termed MMA (MIM 251000). Patients with isolated MMA typically present with recurrent vomiting, respiratory distress, progressive alteration of consciousness, overwhelming illness, deep coma and death. These crises are characterized by lactic acidosis, hypoglycaemia, hyperketonemia, hyperglycinaemia and hyperammonaemia, as well as multiple organ failure 33. Current management approaches for vitamin B12 non‐responsive MMA patients include dietary restriction of propiogenic amino acids, nutritional supplement administration and vigilant monitoring. Liver or combined liver/kidney transplantation has been used to treat those with the most severe clinical manifestations 34. Thus, therapies are necessary to help improve quality of life and prevent liver, renal and neurological complications.

Although the aberrant accumulation of methylmalonic acid may account for multisystem pathological effects including nervous, renal, skin and hepatic dysfunction 35, 36, 37, the molecular mechanisms of methylmalonic acidaemia is not fully obvious. Secondary mitochondrial dysfunction is suggested due to biochemical abnormalities observed in MMA patients during periods of metabolic crisis, which include increased production of lactate and tricarboxylic acid cycle intermediates 38. Recently, abnormal mitochondria morphology was observed in liver and kidney tissues from patients with MMA 38, 39. These abnormalities include mega‐mitochondria with diminutive and disconnected cristae. In addition, reduced oxygen consumption and respiratory chain dysfunction were observed in murine and human studies on MMA and improving mitochondrial function was suggested as a treatment 40. Therefore, we suggest using protein replacement therapy in the form of TAT‐MTS fusion proteins for the treatment of MMA and aimed to determine the role of mitochondrial function in this pathology by checking general mitochondria activity after restoring MCM to MMA patient fibroblasts.

Here, we show that the native and heterologous MTSs fused to the MCM enzyme restored mitochondrial function, enhanced cell viability and reduced MMA levels in MMA patient's fibroblasts. Moreover, this treatment restored albumin and urea secretion in a CRISPR/Cas9‐engineered HepG2 MUT (‐/‐) cell line.

## Experimental procedures

### Cloning of the plasmids encoding the fusion proteins

TAT‐MTSmcm‐MCM and TAT‐∆MTS‐MCM: The plasmid pTAT2.1 [kindly gifted by Dr. Steven F. Dowdy (San Diego School of Medicine, San Diego, CA, USA)] was cut with SacI and NotI, thus obtaining the vector fragment. The full‐length MCM and the MCM lacking its native MTS were generated by PCR using a MCM clone [purchased from OriGene, Rockville, MD, USA, Cat No SC120001 (accession NM_000255)] as a template and a pair of primers covering the whole sequence, including its native MTS (MTSmcm), 5′¬ ACAGCGGAGCTCCTTAAGAGCTAAGAATCAGCTT ‐3′ (forward) and 5′‐ATGACCGCGGCCGCTTATACAGATTGCTGCTTCT ‐3′ (reverse), and a pair of primers covering the mature MCM sequence, 5′¬ ACAGCGGAGCTCCCTACACCAGCAACAGCCC ‐3′, with the same reverse primer, respectively. The PCR fragments were cut with SacI and NotI and ligated with the vector fragment, thus obtaining the plasmids encoding the TAT‐MTSmcm‐MCM and the TAT‐∆MTS‐MCM fusion proteins.

TAT‐MTSlad‐MCM and TAT‐MTScs‐MCM: The plasmid encoding the TAT‐∆MTS‐MCM was cut with NcoI and SacI to remove the TAT sequence, thus obtaining a vector fragment. The heterologous MTSs were obtained as follows: MTSlad was amplified by PCR using the plasmid His‐TAT‐MTSlad‐FXN 41 as a template and the following pair of primers: 5′‐ TAACTTTAAGAAGGAGATATACCATGGGCA‐3′ (forward) and 5′‐ ACAGAGCTCCCTGCGTAAGTTCTCAGAGGCACTG ‐3′ (reverse). MTScs was amplified by PCR using the plasmid TAT‐MTScs‐FXN 41 as a template, and the following pair of primers 5′‐ TAACTTTAAGAAGGAGATATACCATGGGCA‐3′ (forward) and 5′‐ACAGAGCTCCCACTGGCATGCCGGGCTGCAA‐3′ (reverse).

Both PCR products were cut with NcoI and SacI and ligated to the vector fragment, thus obtaining the plasmid encoding the TAT‐MTSlad/MTScs‐MCM fusion proteins.

All plasmids carry a His‐tag at its 5′‐terminus and were confirmed by restriction enzymes and sequencing analyses.

#### Cell culture

Fibroblasts from MMA patients (GM01673, GM00050 cells), both mut(0) cells, were obtained from Coriell Cell Repositories (Camden, NJ, USA) and grown in the recommended medium (Eagle's Minimum Essential Medium with Earle's salts) supplemented with 10% hyclone FBS, 2 mM L‐glutamine, 100 U/ml penicillin and 100 μg/ml streptomycin (Biological Industries, Beit Haemek, Israel). 346 MMA patient fibroblasts were obtained from the Department of Genetic and Metabolic Diseases, Hadassah medical centre, and grown in the same medium. All cell lines were grown at 37°C in humidified atmosphere of 5% CO_2_.

#### Protein expression and purification

E. coli BL21‐CodonPlus (λDE3), Rossetta or HMS competent cells, transformed with plasmids encoding the fusion proteins, were incubated at 37°C in a saline lactose broth (SLB medium) containing kanamycin (50 μg/ml), tetracycline (12.5 μg/ml) and chloramphenicol (34 μg/ml). At an OD600 of 0.2–0.3, 0.1% glycerol and 0.1 mM potassium glutamate were added to the culture and subjected to heat‐shock for 20–30 min. at 42°C. Afterwards, the bacteria were grown at 37°C until an OD600 of 0.8. Protein expression was induced by adding isopropyl‐β‐D‐thiogalactopyranoside (IPTG, for final concentrations see Table 1). After 18 hrs of incubation at 12°C, the cells were harvested by centrifugation (2000 g for 20 min. at 4°C).

**Table 1:** Growth conditions and concentration of the purified TAT‐MTS‐MCM fusion proteins

MTS	Host	IPTG (mM)	Growth conditions	Final concentration mg/ml
MCM	Codon+	0.05	12°C O.N.	0.73
CS	Codon+	0.05	12°C O.N.	2.7
LAD	Rossetta	0.05	12°C O.N.	0.43
Δ	Rossetta	0.05	12°C O.N.	0.75

MTS, mitochondrial targeting sequence; CS, citrate synthase; lad, lipoamide dehydrogenase; Codon+, E. coli BL21‐CodonPlus (λDE3); Rosetta, BL21 derivatives (λDE3) host strain; IPTG, isopropyl‐β‐D‐thiogalactopyranoside.

For the purification procedure, bacteria pellets from 4‐L culture of expressing cells were disrupted using a Microfluidizer (Microfluidics) in binding buffer [(25 mM Tris‐HCl pH8.0, 0.2 M NaCl, 10% glycerol, 5 mM β‐mercaptoethanol, 1 mM phenylmethylsulphonyl fluoride (PMSF) containing 200 ng DNAse]. The suspensions were clarified by centrifugation (24,000 g for 1 hr at 4°C), and imidazole (Sigma‐Aldrich, St. Louis, MO, USA) was added to a final concentration of 10 mM. The supernatants containing the fusion proteins were loaded onto pre‐equilibrated (in binding buffer) HiTrap Chelating HP columns (Amersham Pharmacia Biotech, Uppsala, Sweden). Columns were washed by stepwise addition of increasing imidazole concentrations. Finally, the target proteins were eluted with elution buffer (binding buffer added with 250 mM imidazole). All purification procedures were carried out using the FPLC system ÄKTA (Amersham Pharmacia Biotech). Imidazole was removed by transferring the purified proteins to PBS using PD‐10 desalting columns (GE Healthcare, Piscataway, NJ, USA). Aliquots of the proteins were kept frozen at −80°C until use. Table 1 summarizes the expression conditions of the various TAT‐MTS‐MCM fusion proteins.

### Characterization of the fusion proteins

#### Determination of protein concentration

Protein concentration was measured according to the Bradford method, using the Bradford reagent and a standard curve of BSA. Protein concentration was determined at a wavelength of 595 nm.

#### Separation of proteins by electrophoresis

Samples from the various protein fractions (5–20 μg protein/lane) were loaded on 12% (w/v) SDS‐PAGE gels.

#### Western blot analysis

Samples were separated on 12% SDS‐PAGE gels. The proteins were then electrotransferred onto Immobilon‐P transfer membrane (Millipore, Millipore, Bradford, MA, USA). Western blot analysis was performed using anti‐MCM (Abcam, Cambridge, Mass, USA) dilutions of 1:1000, anti‐His (Amersham Pharmacia Biotech) dilutions of 1:30,000, anti‐albumin (R&D systems, Minneapolis, MN, USA) dilution of 1:1000 and anti‐PDH E1‐α (Cell signaling technology, Boston, MA, USA) to identify the relevant proteins. Band visualization was performed using an enhanced chemiluminescence kit (EZ ECL, Biological Industries).

#### Isolation of mitochondria

Mitochondria were isolated using a differential centrifugation. Cells were homogenized in buffer A (320 mmol/l sucrose, 5 mmol/l Tris‐HCl, 2 mmol/l EGTA, pH 7.4) and centrifuged for 3 min. at 2000 g to remove nuclei and cell debris. The supernatant obtained was centrifuged for 10 min. at 12,000 g at 4°C to pellet the mitochondria. The mitochondrial pellet was washed again twice with buffer A and kept at −80°C until use.

#### Delivery of the fusion protein into cells and its processing within the mitochondria

Cells were plated in three T‐75 flasks. When the cells reached 90% confluence, the medium was replaced with fresh medium containing 100 μg/ml of each TAT‐MTS‐MCM fusion protein, for 3 hrs. After incubation, the cells were washed with phosphate‐buffered saline (PBS), trypsinized, pelleted and kept at −80°C until use. The pellets were re‐suspended in cell lysis buffer (Biological Industries, Beit‐Haemek, Israel) containing 1 mmol/l PMSF, kept on ice for 10 min. and centrifuged at 15,000 g for 10 min. The supernatants were analysed by Western blot using anti‐MCM and anti‐PDH E1‐α antibodies (as described above).

#### ATP levels

15 × 10^3^ cells per well were cultured for 48 hrs in a glucose‐free medium (added with 5 mM galactose) in a black 96‐well plate. ATP levels were measured using the ATPLite luminescence‐based assay according to the manufacturer's instructions (Perkin Elmer, Waltham, MA, USA) and are expressed as levels relative to control patients’ cells, that is not treated with any of the fusion proteins (PBS only added).

#### Oxygen consumption

Oxygen consumption rate (OCR) was measured using an XF24 extracellular flux analyser (Seahorse Biosciences, North Billeric, MA, USA).

#### Mitochondrial membrane potential

Mitochondrial content and mitochondrial membrane potential were estimated using, respectively, MitoTracker Green FM (MTG) (Molecular Probes, Eugene, OR, USA) and tetramethylrhodamine ethyl ester (TMRE) (Abcam). MitoTracker Green was added to the existing medium to a final concentration of 200 nM, and the cells were incubated for 45 min. at 37°C, 5% CO_2_. 30 min. before the end of incubation time, 20 μM Carbonyl cyanide‐4‐(trifluoromethoxy) phenylhydrazone (FCCP) was added as a control. TMRE was added successively to a final concentration of 200 nM, and the cells were incubated for an additional 20 min. at 37°C, 5% CO_2_. Medium was removed and, after rinsing once with PBS for MTG or 0.2% BSA in PBS for TMRE, replaced with 100 μl PBS. The plate was read at 37°C, fluorescence emission wavelengths (λex 485 nm), fluorescence excitation wavelengths (λem 528 nm) for MTG and λex 485 nm, λem 590 nm for TMRE.

#### Cell viability tests

Cells were plated in a 96‐well plate (15 × 10^3^ cells per well) in 100 μl of glucose‐free medium (added with 5 mM galactose) for 24 hrs. The following day, 15 μg/ml of the fusion proteins was added for 72 hrs. Mitochondrial isolation buffer alone was used as control. Cell viability was assayed using CellTiter‐Blue^®^ (Promega, Madison, WI, USA), a fluorescence‐based assay, according to the manufacturer's manual.

### MMA levels

Cells were cultured for 24 hrs in a glucose‐free medium containing 1.25 μM Vitamin B12, an essential cofactor of MCM and 5 mM galactose (Sigma‐Aldrich). MMA levels were determined by a MMA ELISA kit (EIAab, China), according to the manufacturer's instructions *via* a calibration curve. Cells were suspended in sample diluent and lysed by 2‐hr incubation at −80°C.

### CRISPR/Cas9 technology

CRISPR technology was used to knockout the MUT gene in HepG2 cells:

A null mutation was introduced into the MUT gene, using a gRNA to target its ATG start site, to maximize disruption of the wild‐type protein.

Oligos were designed using the Feng Zhang laboratory's Target Finder (http://crispr.mit.edu//). High score sgRNA was chosen (hg19_dna. range = Chr6: 49427158‐49427180) and corresponding oligos were ordered from IDT in phosphorylated form: (Forward: 5phos CACCGCTGATTCTTAGCTCTTAACA; Reverse 5phos AAACTGTTAAGAGCTAAGAATCAGC).

Addgene target sequence cloning protocol (https://www.addgene.org/crispr/zhang) was followed to clone the gRNAs into a pX330 plasmid; the oligos were annealed and ligated with BbsI linearized pX330 vector. Ligated plasmid was transformed into DH5α bacteria. Successful gRNA insertion was confirmed by Sanger sequencing with the U6 promoter primer 5′‐ CAAGGCTGTTAGAGAGATAATTGG A‐3′.

The plasmid was cotransfected with GFP‐expressing plasmid into the HepG2 cells using the ViaFect reagent (Promega). Cells were sorted based on GFP fluorescence 2 days post‐transfection and seeded to receive single‐cell‐derived colonies.

DNA of colonies was extracted and verified for mutations by PCR followed by MseI restriction. The specific primers spanning the gRNA‐mediated DSB were as follows:

Forward primer 5′‐GAAGAAGAGGGGCCAAAGTT‐3′.

Reverse primer 5′‐CCTGGAGCCTGATGATTCTT‐3′.

The MseI site is close to the DSB and is absent when mutation occurs.

In addition to confirm the loss of MCM protein, protein extractions from the MCM null colonies were analysed by Western blot using anti‐MCM antibody.

### Urea determination

The levels of secreted urea were determined using the Clinical Chemistry Analyzer Cobas (cobas^®^ Liat, Roche Diagnostics).

### Statistics

The one‐sample *t*‐test was performed when indicated.

## Results

### Construction, expression and purification of the TAT‐MTS‐MCM fusion proteins

Previously, we efficiently targeted the human frataxin protein into the mitochondria *via* heterologous MTSs 41. In the current research, we aimed to explore whether mitochondrial protein targeting *via* heterologous MTSs could also be used for the human MCM enzyme. We used heterologous MTSs of human, nuclear‐encoded mitochondrial proteins that are classical MTS sequences to target the human MCM protein into the mitochondria, in the form of TAT‐MTS fusion proteins. We used the MTSs of lipoamide dehydrogenase (TAT‐MTSTlad‐MCM) 21, 22, citrate synthase (TAT‐MTScs‐MCM) 41, the native MTS of MCM (TAT‐MTSmcm‐MCM) and a fusion protein lacking a MTS (TAT‐MTS∆‐MCM). MCM is a large protein 87 kD; currently, no protein of this size was targeted to the mitochondria, in the form of a TAT fusion protein system.

**Figure 1:**
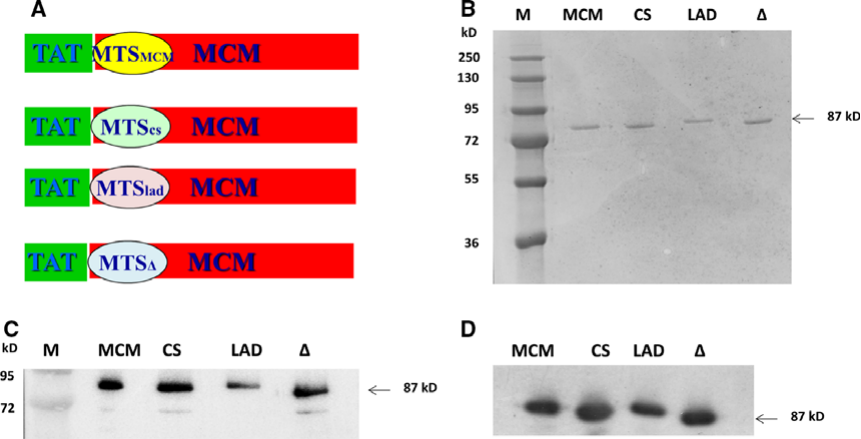
Characterization of the TAT‐MTS‐MCM fusion proteins (**A**) Schematic presentation of the various TAT‐MTS‐MCM fusion proteins. MCM: methylmalonyl‐CoA Mutase; MTS: mitochondrial translocation sequence; cs: Citrate synthase; lad: LAD. (**B**) SDS‐PAGE of the purified fusion proteins. MCM: TAT‐MTSmcm‐MCM, CS: TAT‐MTScs‐MCM and LAD: TAT‐MTSlaMCM. ∆: TAT‐MTS∆‐MCM. (**C‐D**) Western blot analysis of the purified fusion proteins using an anti‐MCM antibody (**C**) and anti‐His antibody (**D**). Arrows indicate the TAT‐MTS‐MCM fusion proteins, at a size of ~87 kD.

All coding sequences were under the control of the T7 promoter, and all plasmids were cloned with His−tag at the 5′−terminus of the coding sequence. Clones were confirmed by restriction enzymes and sequencing analyses. Figure 1A demonstrates the schematic structure of the various TAT‐MTS‐MCM fusion proteins.

Expression of the fusion proteins was performed in E. coli hosts. The expression host and conditions for expression were calibrated for each of the TAT fusion proteins, by changing several parameters, including the concentration of the inducer (IPTG) and length of induction growth conditions (*i.e*. temperature, addition of chemicals; Table 1). Codon+ bacteria were chosen for TAT‐MTScs‐MCM and TAT‐MTSmcm‐MCM, while Rossetta bacteria were chosen for TAT‐MTSTlad‐MCM and TAT‐MTS∆‐MCM fusion proteins (Fig. S1A‐D). Upon expression, bacterial cells were disrupted and cellular subfractions were prepared, separating the soluble and insoluble sub fractions. Analysis was performed for the whole‐cell bacteria and soluble fraction on SDS‐PAGE gels and by Western blots analyses using anti−His antibodies to examine whether the fusion protein was expressed and reaches the soluble subcellular fraction. (Fig. S2A‐F). The goal was to obtain high expression levels of the different TAT fusion proteins in the soluble subfraction of the expressing bacteria, for future purification.

Next, the various fusion proteins were highly purified using affinity chromatography. The soluble fraction of each fusion protein, consisting on a different MTS sequence, was loaded on a Ni‐chelating column, followed by multiple washing steps with increasing concentrations of imidazole and finally eluted at a high concentration of imidazole (Fig. S3A‐D).

To characterize the fusion proteins, the purified fusion proteins were analysed by SDS‐PAGE and Western blot analysis using anti‐MCM (Fig. 1C) and anti‐His antibodies (Fig. 1D). As demonstrated in Figure 1B–D, purified fusion proteins showed a major band at the expected size (approximately 87 kD). A slight size variation is observed among the fusion proteins, resulting from the variation in length of the various MTS polypeptides or its absence.

### Internalization of TAT−MTS−MCM fusion proteins to mitochondria and its processing

**Figure 2:**
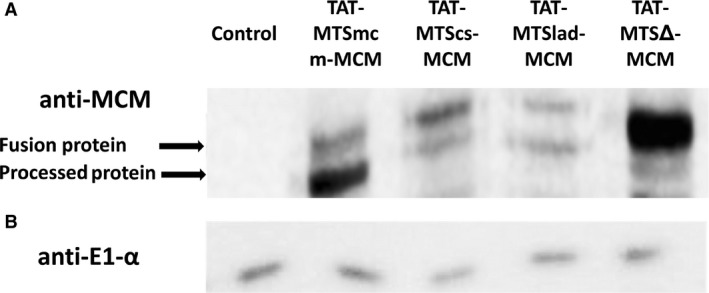
Mitochondrial internalization and processing of TAT−MTS−MCM fusion proteins. (**A**) Western blot analysis, using anti‐MCM antibodies, of isolated mitochondria from GM01673 patient fibroblasts, treated with 100 μg/ml of the different fusion proteins for 3 hrs. Equal amounts (in μg protein) of isolated mitochondria treated with the various fusion proteins were loaded onto the gel. Upper arrow indicates the fusion proteins and lower arrow indicates the processed proteins. (**B**) The purity of the mitochondrial fractions was determined using pyruvate dehydrogenase antibody (anti E1‐α) as a mitochondrial marker. Control; isolated mitochondria from GM01673 patient fibroblasts, not treated with any of the fusion proteins, and added only with PBS.

MCM is a large protein 87 kD; smaller proteins were successfully targeted to the mitochondria in the form of TAT‐MTS fusion proteins. However, no protein this size was reported to reach the mitochondria by this delivery approach. In order to test the ability of the TAT‐MTS‐MCM fusion proteins to reach the mitochondria and undergo processing within intact cells, GM01673, 673 MMA patient fibroblasts, carrying a stop codon mutation for the MCM protein, were incubated for 3 hrs with 100 μg/ml of the various MCM fusion proteins. Afterwards, subcellular fractions were prepared, to separate the mitochondria and the cytosol. Samples were then analysed by Western blot for the presence of the MCM−based fusion proteins using anti−MCM antibodies (Fig. 2A). The purity of the mitochondrial fractions was determined using pyruvate dehydrogenase antibody (anti E1‐α, Fig. 2B) as a mitochondrial marker (and as a loading control), and GAPDH antibody as a cytosol marker (data not shown). The anti‐MCM staining confirmed the existence of the fusion proteins within the mitochondria. Moreover, this staining demonstrated that the MCM fusion proteins underwent processing, as evident by the appearance of an additional product, smaller in size that reacted with the specific antibody (marked as ‘Processed Protein’, Fig. 2A). TAT‐MTSmcm‐MCM undergoes processing better than the heterologous MTSs. The TAT‐MTS∆‐MCM fusion protein that lacks any MTS was the only fusion protein that did not underwent any processing as expected (Fig. 2A). TAT‐MTS∆‐MCM reached the mitochondria most probably due to the TAT sequence, which allows its crossing through biological membranes, including mitochondrial membranes. However, TAT fusion proteins targeted to mitochondria not by the canonical mitochondrial import pathway do not remain there 19.

### Effect of mitochondrial targeted MCM fusion proteins on mitochondrial function

**Figure 3:**
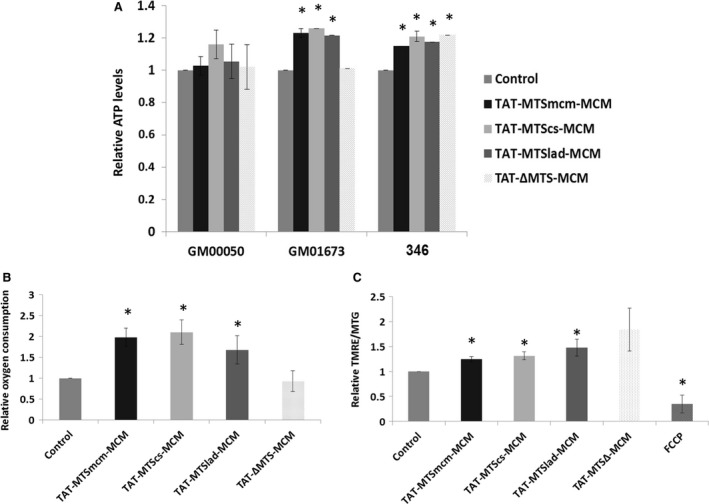
TAT‐MTS‐MCM fusion proteins increase mitochondrial ATP levels, oxygen consumption and mitochondrial membrane potential in MMA patient's cells. (**A**) ATP levels of 346, GM01673 and GM00050 MMA patient fibroblasts grown in glucose‐free medium (added with 5 mM galactose) for 48 hrs. 15 × 10^3^ cells were incubated with 10 ug/ml of each of the fusion protein for 6 hrs and then analysed for total cell ATP using ATPLite luminescence‐based assay. Results represent mean ± S.E.M.,* n* = 3, *P ≤ 0.05**.** (**B**) Oxygen consumption of GM01673 fibroblasts grown in glucose‐free medium (added with 5 mM galactose) for 48 hrs. 15 × 10^3^ cells were incubated with 10 μg/ml of the various fusion proteinsfor 6 hrs. Oxygen consumption was determined using Seahorse ExtracellularFlux (XF) Analyzer. Results represent mean ± S.E.M.,* n* = 3, *P ≤ 0.05. (**C**) Mitochondrial membrane potential of GM01673 fibroblasts grown in glucose‐free medium (added with 5 mM galactose) for 48 hrs. 15 × 10^3^ cells were incubated with 15 μg/ml of the tested fusion protein for 6 hr, and 1 hr before the end of incubation time, 200 nM MitoTracker Green FM was added. In addition, 30 min. before the end of incubation time, 20 μM FCCP was added as a control, and 20 min. before the end of incubation time, 200 nM TMRE was added. Results represent mean ± S.E.M.,* n* = 4, *P ≤ 0.05.

Although the aberrant accumulation of methylmalonic acid may account for multisystem pathological effects, the role of mitochondria in this process is not clear. To this end, we determine whether mitochondrial targeted MCM fusion proteins could affect common mitochondrial functions by measuring ATP produced by OXPHOS, oxygen consumption and mitochondrial membrane potential. GM01673, GM00050 or 346 MMA patient fibroblasts were cultured for 48 hrs in a glucose‐free, OXPHOS‐dependent medium (added with 5 mM galactose), supplemented with dialysed serum and 1.25 μM Vitamin B12, an essential cofactor of the MCM enzyme. Mitochondrial ATP levels were determined 6 hrs after incubation with 10 μg/ml of each TAT‐MTS‐MCM fusion proteins. As shown in Figure 3A, a significant increase of 21–25% in ATP levels was observed in 346 MMA patient cells by all fusion proteins, while TAT‐MTScs‐MCM and TAT‐MTS∆‐MCM treatment resulted in the largest increase. In GM01673 MMA patient fibroblasts, an increase of 14–21% was observed in ATP levels, upon treatment. TAT‐MTScs‐MCM treatment demonstrated the highest increase. Interestingly, treatment with TAT‐MTS∆‐MCM did not affect ATP levels, implying that the processing phase is important for MCM activity. Treatment of GM00050 MMA patient fibroblasts with the various TAT‐MTS‐MCM fusion proteins although not significant resulted in increased activity. TAT‐MTS∆‐MCM enhanced mitochondrial ATP production in 346 patient fibroblasts, but not in GM01673 patient fibroblasts. GM01673 MMA patient cells are classical mut (0) cells; however, 346 cells, although obtained from a MMA patient, are not fully characterized and are not mut (0) cells (data not shown). Therefore, the exogenous TAT‐MTS∆‐MCM may interact with the endogenous MCM to elevate ATP production.

Oxygen consumption was determined in GM01673 MMA patient fibroblasts 6 hrs after incubation with 10 μg/ml of each TAT‐MTS‐MCM fusion protein. As shown in Figure 3B, significant increase of 50–102% was observed in oxygen consumption upon treatment. Treatment with TAT‐MTScs‐MCM resulted in the highest increase in oxygen consumption, while again treatment with TAT‐MTS∆‐MCM showed reduced oxygen consumption, as compared to untreated cells.

Mitochondrial membrane potential was determined 6 hrs after incubation with 15 μg/ml of each TAT‐MTS‐MCM fusion protein. The TMRE/MTG ratio was measured. As shown in Figure 3C, significant increase of 24–47% was observed in mitochondrial membrane potential upon treatment, indicating an increase in general mitochondrial activity. Treatment with TAT‐MTSlad‐MCM resulted in the highest change in mitochondrial membrane potential. Although treatment with TAT‐MTS∆‐MCM enhanced mitochondrial membrane potential, this change was not significant. FCCP, a known uncoupler of OXPHOS, reduce mitochondrial membrane potential as expected (Fig. 3C).

### Effect of mitochondrial targeted MCM fusion proteins on cell viability

**Figure 4:**
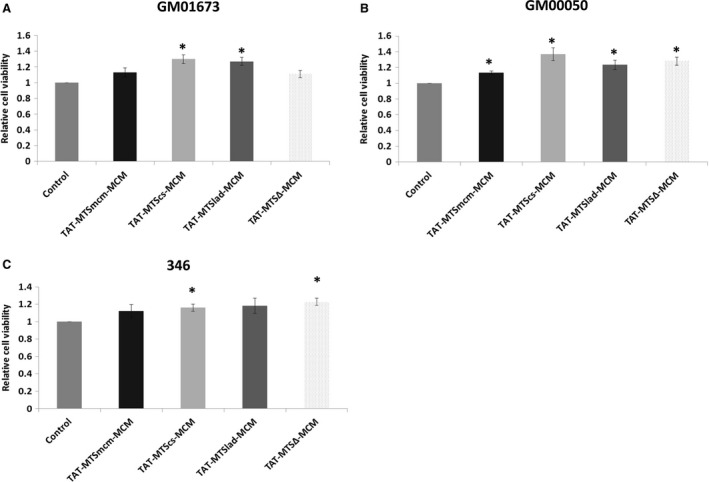
TAT‐MTS‐MCM fusion proteins increase cell viability of MMA patient's cells. Cell viability levels of GM01673 (**A**), GM00050 (**B**) and 346 (**C**) patient fibroblasts grown in glucose‐free medium for 24 hrs. 15 × 10^3^ cells were incubated with 15 μg/ml of the various fusion proteins for 72 hrs and then analysed for cell viability. Mitochondrial isolation buffer alone was used as control. Cell viability was assayed using CellTiter‐Blue^®^ (Promega) a fluorescence‐based assay, according to the manufacturer's manual. Results represent mean ± S.E.M.,* n* = 3, *P < 0.05.

In order to determine whether delivery of TAT‐MTS‐MCM fusion proteins into MMA patient cells could affect cell viability, GM01673, GM00050 or 346 patient fibroblasts were cultured for 24 hrs in an OXPHOS‐dependent medium. Cell viability was determined 72 hrs after incubation with 15 μg/ml of each fusion protein. As shown in Figure 4, a significant increase of 13–30% in cell viability was observed in GM01673 patients’ cells (Fig. 4A), 11–36% in GM00050 cells (Fig. 4B) and 12–22% in 346 (Fig. 4C) fibroblasts, with all fusion proteins. Again, treatment with TAT‐MTScs‐MCM fusion protein resulted in the highest improvement (27%) in cell viability, whereas TAT‐MTS∆‐MCM treatment showed reduced activity in GM01673 cells as compared to untreated cells.

### Effect of mitochondrial targeted MCM fusion proteins on MMA levels

**Figure 5:**
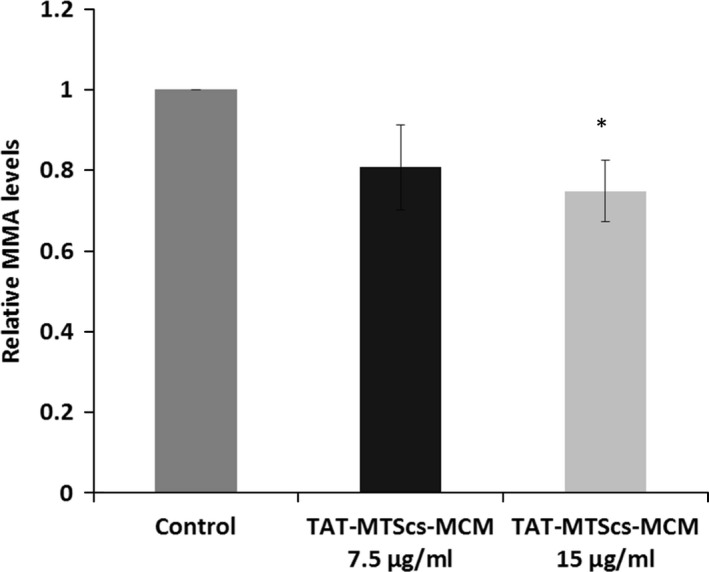
TAT‐MTS‐MCM fusion proteins reduce MMA levels in MMA patient's cells. MMA ELISA analysis of 2 × 10^6^ GM01673 fibroblasts grown in glucose‐free medium for 24 hrs. Afterwards, cells were incubated with either 7.5 or 15 μg/ml of TAT‐MTScs‐MCM for 48 hrs and then analysed for total cell MMA levels. Results represent mean ± S.E.M.,* n* = 4, *P < 0.05.

The major symptom of MMA pathology is elevated MMA levels, which may account for multisystem pathological effects 35, 37. In order to determine whether delivery of TAT‐MTS‐MCM fusion proteins into the mitochondria of MMA patient cells could reduce MMA levels, GM01673 patient fibroblasts were cultured for 24 hrs in an OXPHOS‐dependent medium. Methylmalonic acid levels were determined in whole‐cell lysates using an ELIZA kit 48 hrs after incubation with 7.5 or 15 μg/ml of TAT‐MTScs‐MCM. As shown in Figure 5, a 25% significant reduction in MMA levels was observed after treatment with the TAT‐MTScs‐MCM fusion protein (at 15 μg/ml).

### Effect of TAT‐MTS‐MCM fusion proteins on liver secretion

**Figure 6:**
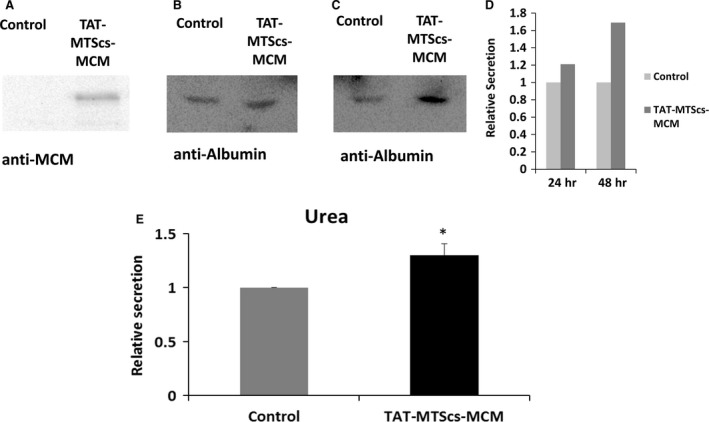
TAT‐MTS‐MCM fusion proteins increase albumin and urea secretion from MCM (‐/‐) HepG2 cells. (**A‐C**) Secreted albumin levels in the growth medium. 5 × 10^5^ HepG2 MCM (‐/‐) cells, confirmed to lose the MCM protein expression (**A**), were grown in OXPHOS‐dependent medium for 24 hrs. Afterwards, cells were incubated with 15 μg/ml of TAT‐MTScs‐MCM for 24 (**B**) or 48 hrs (**C**), growth medium was collected and centrifuged for 5 min. 500 g. Albumin levels were determined by Western blot analysis, using anti‐albumin antibodies. Equal aliquots of the growth medium were loaded. (**D**) Quantitate data of B&C. (**E**) Secreted urea levels in the growth medium. 5 × 10^5^ HepG2 MCM (‐/‐) cells were grown in complete medium supplied for 24 hrs. Afterwards, cells were incubated in OXPHOS‐dependent medium added with 15 μg/ml of TAT‐MTScs‐MCM for 24 hrs. Then, growth medium was collected and centrifuged for 5 min. 14,000 g, and the remaining cells were lysed. Urea levels were determined by a Cobas analyser. Results represent mean ± S.E.M.,* n* = 3, *P < 0.05. Control; HepG2 MCM (‐/‐) cells not treated with any of the fusion proteins, and added only with PBS.

The effects of TAT‐MTS‐MCM fusion proteins were all tested in cultures of fibroblast obtained from MMA patients. However, as the main organ affected in MMA pathology is the liver 42, we aimed to produce a liver cell line which lacks MCM expression to mimic MMA liver pathology. Recently, Erlich *et al*. have shown that mitochondrial function is essential for secretion of mediators from mast cells 43. Therefore, our hypothesis was that secretion in MCM‐mutated liver cells could be impaired. To check this hypothesis, we knocked out the MUT gene from HepG2 cells, an accepted model for albumin secretion 44, using the CRISPR technology. Deletion of the MUT gene was confirmed by Western blot analysis with anti‐MCM antibodies (Fig. 6A, control line). MCM protein levels were not detected in HepG2 MCM (‐/‐) cells. Next, the levels of albumin were determined by Western blot analysis in the growth medium of the HepG2 mut(‐/‐) cells (OXPHOS medium) treated for 24 hrs (Fig. 6B) or 48 hrs (Fig. 6C) with 15 μg/ml of TAT‐MTScs‐MCM, compared to untreated cells. As shown in Figure 6B–C, the levels of secreted albumin were increased following treatment with TAT‐MTScs‐MCM (21% for 24 hrs, 69% for 48 hrs; Fig. 6D). In addition, it is well‐established that urea is secreted from liver cells. Therefore, the levels of urea in the growth medium were also determined by Covas analysis after treatment for 48 hrs with TAT‐MTScs‐MCM fusion protein (samples were normalized to the protein concentration of the lysed cells). As shown in Figure 6E, the levels of secreted urea were increased by 29% following treatment with the TAT‐MTScs‐MCM fusion protein. To conclude, restoring MCM activity in liver cells may affect major functions of the liver such as albumin and urea secretion.

## Discussion

Currently, there is no cure for MMA pathology and the main treatment for MMA patients is dietary restriction of propiogenic amino acids and carnitine supplementation 45. Liver or combined liver/kidney transplantation has been used to treat those with the most severe clinical manifestations 42. Thus, therapies are necessary to help improve quality of life and prevent liver, renal and neurological complications. Several new therapies have been suggested for the treatment of mitochondrial disorders such as allosteric activation of OXPHOS complexes, scavenging toxic intermediates, stimulation of mitochondrial biogenesis and protein replacement 9. In addition to mitochondrial dysfunction observed in biopsies from MMA patients, there is also a need to moderated MMA levels, a key factor in this pathology.

Here, we show that the native and heterologous MTSs fused to the MCM protein are easily produced and highly purified, although being a large protein (Fig. S1–S3). In addition, these fusion proteins successfully undergo processing to obtain the native MCM enzyme within the mitochondria (Fig. 2). Furthermore, treatment with these fusion proteins restored mitochondrial function measured by ATP levels, oxygen consumption and mitochondrial membrane potential in MMA patient's fibroblasts (Fig. 3). Moreover, cell viability of the patient's fibroblasts was enhanced after treatment with the fusion proteins (Fig. 4) and reduced MMA levels were also observed (Fig. 5). In addition, this treatment enhanced albumin and urea secretion in a CRISPR/Cas9‐engineered HepG2 MUT (‐/‐) liver cell line (Fig. 6).

We were the first to use TAT‐MTS fusion proteins to target a functional protein to the mitochondria for the pre‐clinical treatment of LAD deficiency 21, 22. However, TAT‐MTS fusion proteins have been used successfully for the pre‐clinical treatment of mitochondrial complex I deficiency 46, cytochrome c oxidase deficiency 47 and Friedreich's ataxia 41, 48. These studies clearly show the ability of the approach to target proteins into the mitochondria both *in vitro* and *in vivo*. In addition, the targeted protein could be assembled into large complexes within the mitochondria and only a small amount of replaced mitochondrial protein can restore proper function.

In this study, we examined whether TAT‐mediated enzyme replacement therapy could be a potential treatment for MMA and determined the role of mitochondria in this pathology. In addition to the native MTS of the MCM enzyme, we used heterologous MTSs of classical mitochondrial proteins in order to explore whether these fusion proteins could better restore MCM function. Surprisingly for a large protein 87 kD, all TAT‐MTS‐MCM fusion proteins were highly purified in high concentrations as indicated by SDS‐PAGE and Western blot analysis (Table 1 and Fig. S1–S3). TAT‐MTS‐MCM is the largest protein targeted to the mitochondria in the form of TAT‐MTS‐MCM fusion proteins, suggesting that this approach could be relevant even to deficiencies with large defective proteins.

The aberrant accumulation of methylmalonic acid may account for multisystem pathological effects including nervous, renal, skin and hepatic dysfunction 35, 36, 37. However, the molecular mechanism of methylmalonic acidaemia is not fully clear. Recently, an effect on general gene expression was suggested, as high levels of MMA were shown to change the expression of over 500 genes 49. Secondary mitochondrial dysfunction is suggested due to biochemical abnormalities observed in MMA patients 38. Abnormal mitochondria morphology was observed in liver 38 and kidney 39 tissues from patients with MMA, and reduced oxygen consumption and respiratory chain dysfunction were observed in murine and human studies on MMA 50, 51, 52, 53. Therefore, in addition to the depletion in energy production due to the absence of succinyl‐CoA in MMA pathology, elevated MMA levels could also affect expression of mitochondrial proteins and may explain this severe pathology.

We aimed to determine the role of mitochondria in this pathology, by measuring general mitochondria activity after incubation of MMA patient fibroblasts with the fusion proteins. Restoring MCM activity enhanced mitochondrial ATP production with all fusion proteins in 346 patient fibroblasts, with all fusion proteins except for TAT‐MTS∆‐MCM in GM01673 patient fibroblasts (Fig. 3A). In addition, although TAT‐MTSmcm‐MCM processing was superior compared to the other fusion proteins, no benefit in all mitochondrial activities measured for this fusion protein was seen. This could be as only a small amount of replaced enzyme is needed for the correction of the deficit in MCM activity. Furthermore, the different MTSs may affect the cleavage and dimerization of the protein and therefore its activity as well. As the TAT‐MTS‐MCM fusion proteins enhanced various mitochondria characteristics, such as ATP levels, oxygen consumption and mitochondrial membrane potential, we could conclude that the mitochondria play a major role in the pathology of MMA, confirming other reports 30, 31, 42, 43, 44.

Next, we determined whether this increase in mitochondria function following treatment with the fusion proteins could affect cell viability. Our results clearly show that the increase in mitochondrial function correlates with an enhancement in cell viability in all patient cells (Fig. 4). The heterologous MTSs increased cell viability to a greater extent than the native MTS of MCM in these studies. Therefore, together with the mitochondrial function experiments, we could conclude that the use of heterologous MTSs can be beneficial for the treatment of MMA pathology. Furthermore, TAT‐MTScs‐MCM restored mitochondrial functional activities to the greatest extent (Figs 3 and 4).

The major symptom of MMA pathology is elevated MMA levels. We used the most potent fusion protein TAT‐MTScs‐MCM to check whether this treatment could reduce MMA levels in GM01673 patient fibroblast. Treatment with TAT‐MTScs‐MCM reduced total MMA levels close to 25%. Interestingly, other reported treatments 45 based on gene therapy using an adeno‐associated virus serotype 8 vector (AAV8) also show that the levels of MMA are not thoroughly reduced as expected (~40% reduction observed) 54. This is consistent even if the AAV8 is delivered to newborn mice and even after re‐administration of the virus 55. Therefore, the mitochondria may be already irreversibly damaged at the time of the treatment. Thus, treatment at an earlier stage, for example during pregnancy, should be considered.

The main organ affected in MMA pathology is the liver 42. To check whether TAT‐MTS‐MCM fusion proteins could affect liver function such as albumin and urea secretion, we produced a HepG2 MCM (‐/‐) cell line using the CRISPR/Cas9 technology. To our knowledge, this is the first time this technology has been used to study MMA pathology. We used the most potent fusion protein TAT‐MTScs‐MCM to check whether this treatment could increase albumin and urea secretion in liver mutated cells. TAT‐MTScs‐MCM treatment indeed elevated albumin and urea secretion. Therefore, we suggest a role for mitochondrial function and MCM activity in secretion of mediators from the liver.

TAT has the ability to cross the placenta and successfully reach the heart, liver and brain tissues from both the foetus and pups 19. The use of protein therapeutics replacing a protein that is deficient or mutated is recently growing 56, 57, 58. Protein therapeutics have been approved for the use of endocrine disorders (hormone deficiencies) such as diabetes mellitus 59 and growth failure due to GH deficiency 60, haemostasis and thrombosis disorders 61, 62, metabolic enzyme deficiencies such as Gaucher's disease 63 and Pompe disease 64, pulmonary and gastrointestinal tract disorders 65, 66 and more. In addition, there is a wide use of PTDs for tissue‐specific targeting of proteins. For example, p28 has been reported to preferentially target cancer cells and has successfully past phase 1 studies 67. Several other specific PTDs have been identified, including for cardiac cells 68, vascular endothelial cells 69 and dendritic cells 70. However, these studies await clinical trials. Cell‐penetrating peptides using TAT have recently past phase 1 and phase 2 studies 56. The immunogenicity and toxicity of TAT have been evaluated in several studies. TAT fused to albumin was incubated with epithelial cells from skin, lung and intestine, the cells most likely to first come into contact with systemically administered PTD therapeutics. The TAT fusion protein successfully internalized the cells without altering their viability. Furthermore, they failed to induce an innate immune response, as no activation of phosphorylated NFκB, a signalling molecule downstream of toll‐like receptors was observed 71. Likewise, no secretion of epithelial‐specific interleukins IL‐6 and IL‐8 was detected 71. Similar results were seen after incubation of TAT fusion proteins with peripheral blood mononuclear cell lines both *in vitro* and *in vivo* 72. In addition, comprehensive toxicity studies including cell viability, proliferation and leakage of lactate dehydrogenase showed no toxic effects of TAT compared to other PTDs 73, 74. Other advantages of TAT are its high transduction efficiency, little perturbation to the plasma membrane and its ability to transfer the blood–brain barrier 75, 76, which is necessary for the treatment of MMA pathology.

To conclude, the TAT‐MTS‐MCM fusion proteins could be a potential treatment for MMA pathology. The use of heterologous MTSs of classical mitochondrial nucleus‐encoded proteins could be beneficial for targeting the MCM protein into the mitochondria and thus for MMA patients. Furthermore, our results clearly show that mitochondria play a major role in MMA pathology.

## Conflict of interest

The authors confirm that there are no conflict of interests.

## Supporting information


**Fig. S1** Host calibration of the fusion proteins.
**Fig. S2** Expression of the fusion proteins.
**Fig. S3** Purification of the fusion proteins using Ni‐chelating column affinity chromatography.
