# Supplementary material for: TAT‐MTS‐MCM fusion proteins reduce MMA levels and improve mitochondrial activity and liver function in MCM‐deficient cells
Source: J Cell Mol Med. 2017 Dec 19;22(3):1601–13. doi: 10.1111/jcmm.13435 (PMC5824393; doi:10.1111/jcmm.13435)
Supplement: Supplementary file 1 — Fig. S1 Host calibration of the fusion proteins. Fig. S2 Expression of the fusion proteins. Fig. S3 Purification of the fusion proteins using Ni‐chelating column affinity chromatography. [file JCMM-22-1601-s001.pdf]

Supplementary Figure Legends

Fig.S1: Host calibration of the fusion proteins.

A-D: SDS page analysis of the expression in un-induced (un), IPTG induced (in) of the different fusion proteins in various bacteria hosts. TAT-MTSmcm-MCM (A), TAT-MTScs-MCM (B), TAT-MTSlad-MCM (C) and TAT-MTSΔ-MCM (D).

Fig.S2: Expression of the fusion proteins.

A-D: SDS-PAGE showing expression of TAT-MTSmcm-MCM (A), TAT-MTScs-MCM (B), TAT-MTSlad-MCM- (C) and TAT-MTSΔ-MCM (D), in un-induced (un), IPTG induced (in), whole cell extract (WCE) and soluble (sol) in codon + and Rosseta bacteria .

E-F: Western blot analysis of A and B using an anti-His antibodies .

Fig.S3: Purification of the fusion proteins using Ni-chelating column affinity chromatography.

A-D SDS-PAGE analysis of the purification of the fusion proteins following affinity chromatography, using a Ni-chelating column. A: TAT-MTSmcm-MCM, B: TAT-MTScs-MCM, C: TAT-MTSlad-MCM. D: TAT-MTSΔ-MCM

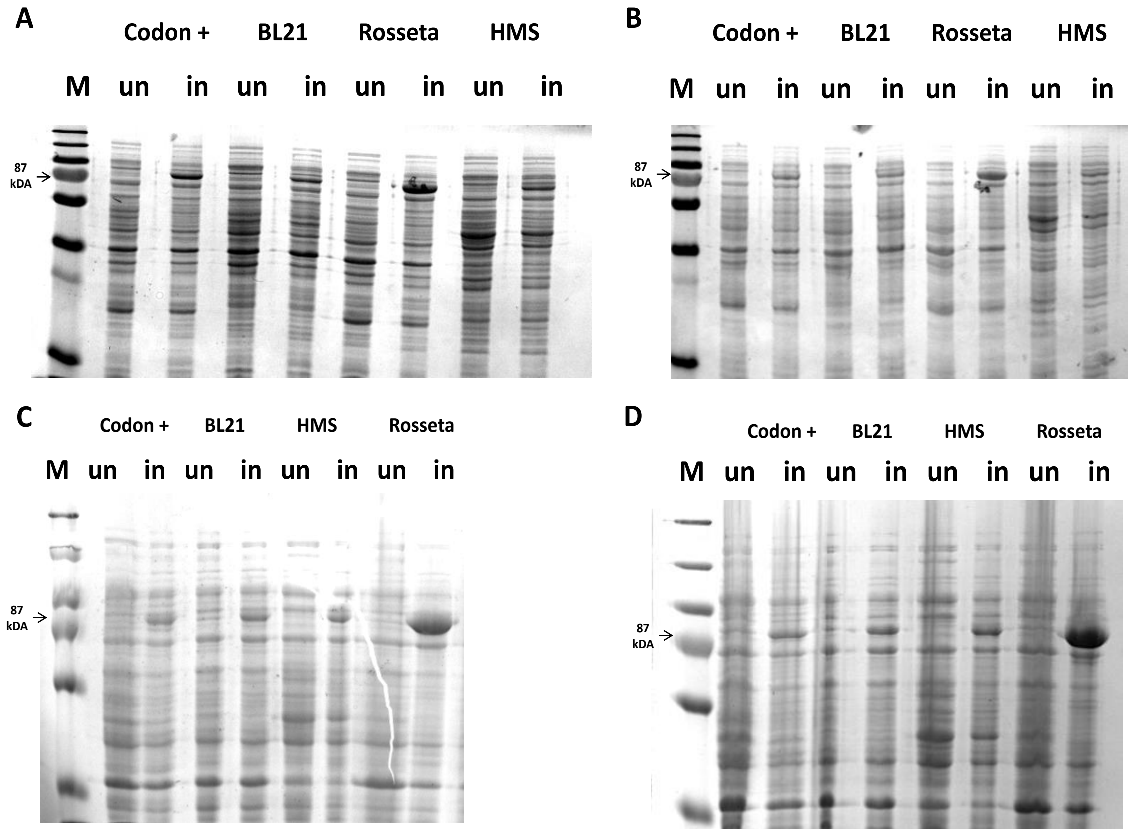
A
Codon + BL21 Rosseta HMS
M un in un in un in un in
87 kDA
B
Codon + BL21 Rosseta HMS
M un in un in un in un in
87 kDA
C
Codon + BL21 HMS Rosseta
M un in un in un in un in
87 kDA
D
Codon + BL21 HMS Rosseta
M un in un in un in un in
87 kDA

Figure S1

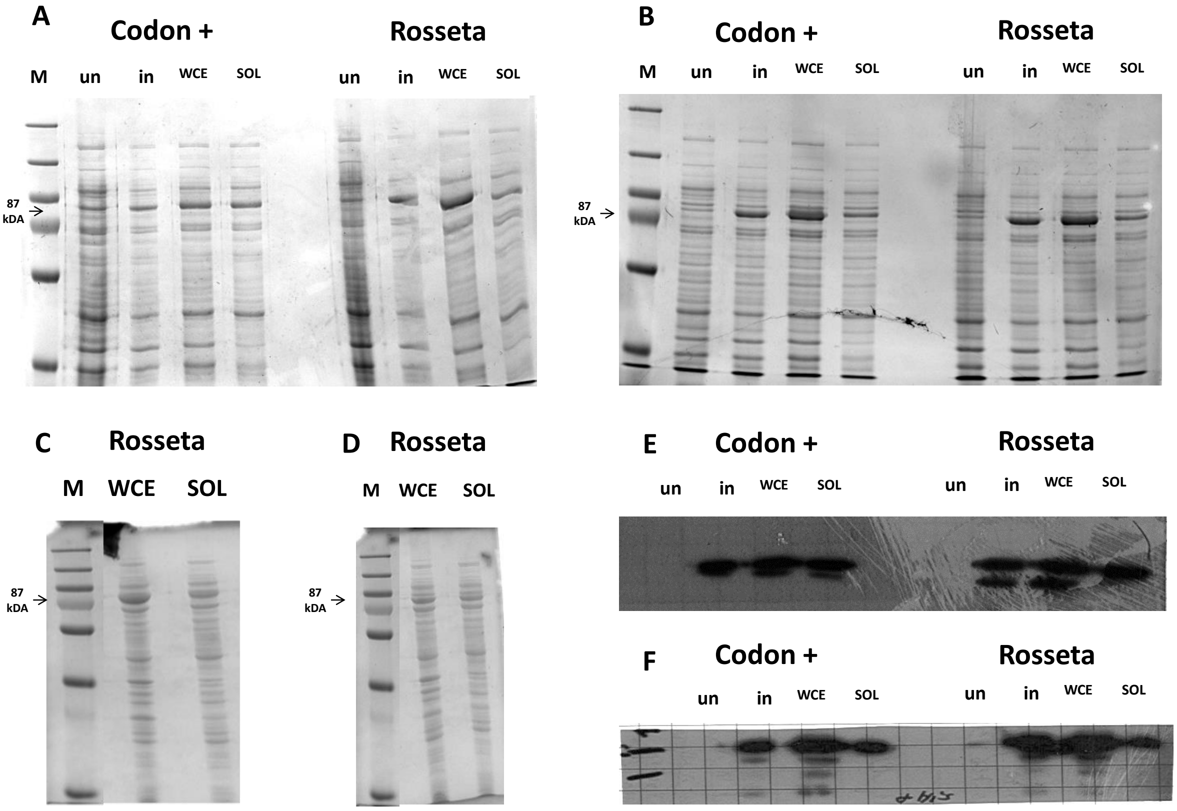
A
Codon +
Rosseta
M
un
in
WCE
SOL
un
in
WCE
SOL
87 kDA
B
Codon +
Rosseta
M
un
in
WCE
SOL
un
in
WCE
SOL
87 kDA
C
Rosseta
M
WCE
SOL
87 kDA
D
Rosseta
M
WCE
SOL
87 kDA
E
Codon +
Rosseta
un
in
WCE
SOL
un
in
WCE
SOL
F
Codon +
Rosseta
un
in
WCE
SOL
un
in
WCE
SOL

Figure S2

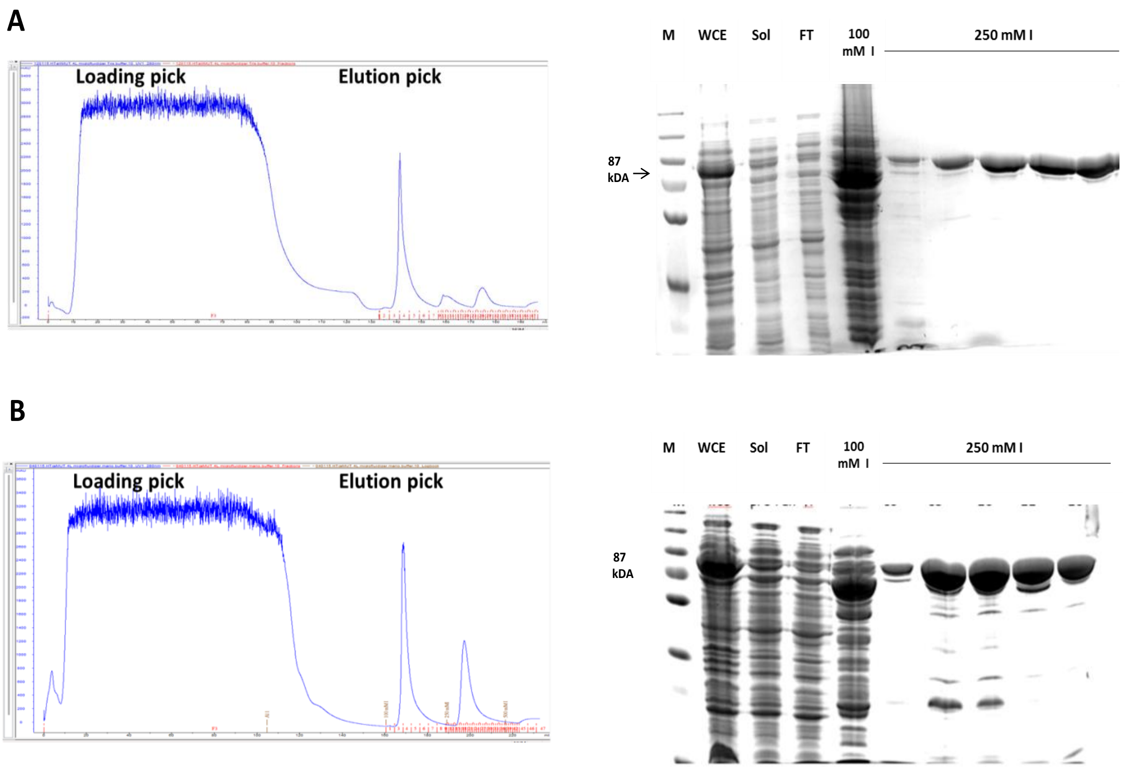
A
Loading pick
Elution pick
M
WCE
Sol
FT
100 mM I
250 mM I
87 kDA
B
Loading pick
Elution pick
M
WCE
Sol
FT
100 mM I
250 mM I
87 kDA

Figure S3

C

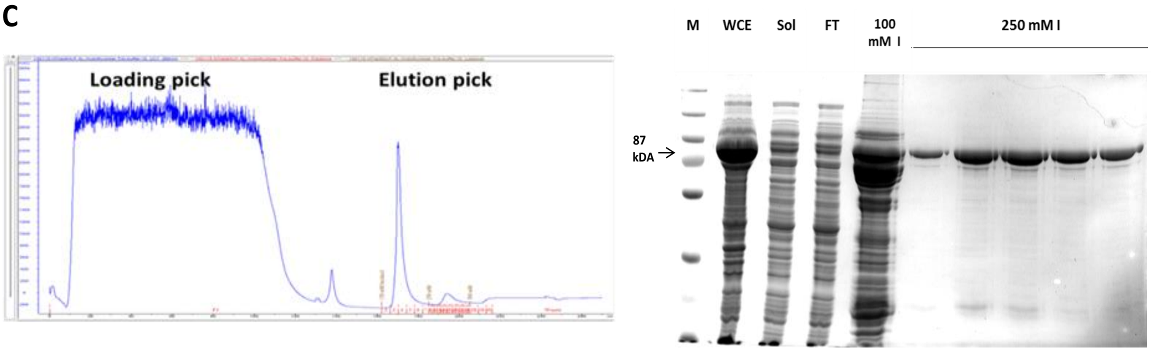

Loading pick
Elution pick
M
WCE
Sol
FT
100 mM I
250 mM I
87 kDA

D

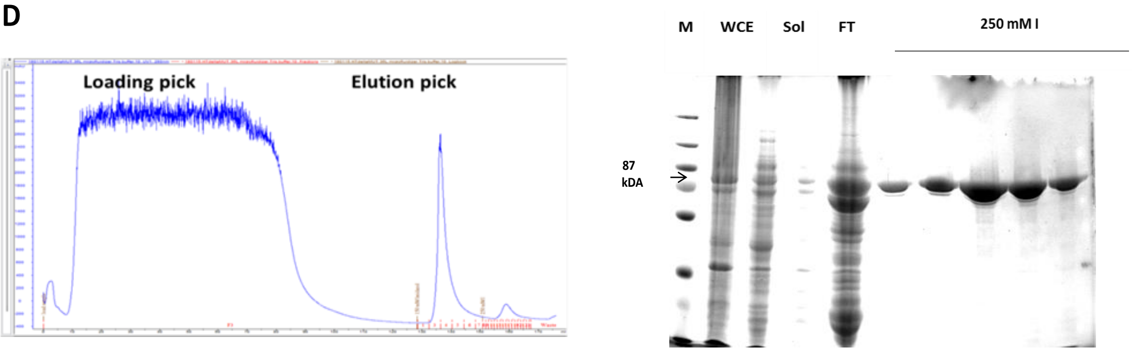

Loading pick
Elution pick
M
WCE
Sol
FT
250 mM I
87 kDA

Figure S3
